# Development, In-Vitro Characterization and Preclinical Evaluation of Esomeprazole-Encapsulated Proniosomal Formulation for the Enhancement of Anti-Ulcer Activity

**DOI:** 10.3390/molecules27092748

**Published:** 2022-04-25

**Authors:** Dibyalochan Mohanty, Ameeduzzafar Zafar, Mohammed Jafar, Atul Kumar Upadhyay, Mohammad Akiful Haque, Jeetendra Kumar Gupta, Vasudha Bakshi, Mohammed M. Ghoneim, Sultan Alshehri, Mohammed Asadullah Jahangir, Mohammed Javed Ansari

**Affiliations:** 1 Department of Pharmaceutics, Anurag University, Hyderabad 500088, India; vasudhapharmacy@cvsr.ac.in; 2 Department of Pharmaceutics, College of Pharmacy, Jouf University, Sakaka 72341, Saudi Arabia; azafar@ju.edu.sa; 3 Department of Pharmaceutics, College of Clinical Pharmacy, Imam Abdulrahman Bin Faisal University, Dammam 34212, Saudi Arabia; mjomar@iau.edu.sa; 4 Department of Biotechnology, Thapar Institute of Engineering & Technology, Patila 147001, India; atul.upadhyay@thapar.edu; 5 Department of Pharmaceutical Analysis, Anurag University, Hyderabad 500088, India; akif963@gmail.com; 6 Institute of Pharmaceutical Research, GLA University, Mathura 281406, India; jk.gupta@gla.ac.in; 7 Department of Pharmacy Practice, College of Pharmacy, Al-Maarefa University, Ad Diriyah 13713, Saudi Arabia; mghoneim@mcst.edu.sa; 8 Department of Pharmaceutics, College of Pharmacy, King Saud University, Riyadh 11451, Saudi Arabia; salshehri1@ksu.edu.sa; 9 Department of Pharmaceutics, Nibha Institute of Pharmaceutical Sciences, Rajgir 803116, India; 10 Department of Pharmaceutics, College of Pharmacy, Prince Sattam Bin Abdulaziz University, AlKharj 11942, Saudi Arabia; javed.pharma@gmail.com

**Keywords:** esomeprazole, proniosomes, Box–Behnken statistical design, pharmacokinetic, Anti-ulcer activity

## Abstract

**Objective:** The present study aimed to develop and optimize esomeprazole loaded proniosomes (EZL-PNs) to improve bioavailability and therapeutic efficacy. **Method:** EZL-PNs formulation was developed by slurry method and optimized by 33 box-Bhekhen statistical design software. Span 60 (surfactant), cholesterol, EZL concentration were taken as independent variables and their effects were evaluated on vesicle size (nm), entrapment efficiency (%, EE) and drug release (%, DR). Furthermore, optimized EZL-PNs (EZL-PNs-opt) formulation was evaluated for ex vivo permeation, pharmacokinetic and ulcer protection activity. **Result:** The EZL-PNs-opt formulation showed 616 ± 13.21 nm of vesicle size, and 81.21 ± 2.35% of EE. EZL-PNs-opt exhibited negative zeta potential and spherical confirmed scanning electron microscopy. EZL-PNs-opt showed sustained release of EZL (95.07 ± 2.10% in 12 h) than pure EZL dispersion. The ex-vivo gut permeation result exhibited a significantly (*p* < 0.05) enhanced flux than pure EZL. The in vivo results revealed 4.02-fold enhancement in bioavailability and 61.65% protection in ulcer than pure EZL dispersion (43.82%). **Conclusion:** Our findings revealed that EZL-PNs formulation could be an alternative delivery system of EZL to enhance oral bioavailability and antiulcer activity.

## 1. Introduction

Esomeprazole (EZL) is an S-isomer of omeprazole and a US Food and Drug Administration (FDA)-approved proton pump inhibitor. It is a strong acid inhibitor and inactive at neutral pH. EZL is used for the treatment of various acid associated diseases like gastric ulcer, Zollinger elision syndrome, gastroesophageal reflux disease and erosive esophagitis [1]. EZL is low water-soluble and highly permeable [2]. It has superior pharmacokinetic profiling over omeprazole in monitoring acid-related ailments [3]. The fact that the stability of esomeprazole decreases with a decrease in pH of media means that the contact of EZL with the acidic content of the stomach leads to degradation of EZL and may reduce the bioavailability [4]. To prevent EZL from acid degradation, enhance the bioavailability and therapeutic efficacy, various formulations has been reported like EZL loaded enteric-coated minitablets [4], gastric resistance pellets [5], buccal adhesive tablets [6] pH-sensitive hydrogels [7] and microspheres [8]. However, these formulations have reported limited absorption and the multiple-unit pellet systems provide some advantages over conventional systems such as by modifying the drug release, content uniformity and weight variation [9,10].

The dosage form is administered through various routes to reach the target site. These carrier systems are being widely used for the delivery of a therapeutic agent into the body. These systems are used to improve the physicochemical and biological characteristics of drugs. There are various colloidal systems reported to improve the stability, therapeutic efficacy and bioavailability of drugs like noisomes [11], nanoparticulate carriers [12,13], liposomes [14], proniosomes [15], proliposomes [16], transferosomes [17] and pharmacosomes [18].

However, liposome and noisome formulations are associated with drawbacks such as physical stability i.e., drug leakage, sedimentation and the fusion of vesicles. These drawbacks can be overcome by the proniosomes (PNs) system [19]. PNs systems have gained attention in recent years due to their superiority over noisome and liposomes systems [20,21]. They are a free-flowing dry powder composed of lipid, surfactant and inert solid material (maltodextrin) and simply rehydrated with water before administration into the body. PNs is a biodegradable, biocompatible and non-immunogenic, and displays flexibility in its structural characterization. It has advantages such as high physical stability, no leakage of an encapsulated drug on prolonged storage, insignificant chemical degradation, ease of transportation, and storage [22,23]. A PNs system also improves the bioavailability of drugs and overcoming the gastro-intestinal tract barrier via transcytosis of M-cells from Peyer’s patches at the GIT lymphatic system [20]. There are various research reports available on PNs to improve the therapeutic efficacy of drugs. Celecoxib loaded PNs were formulated and exhibited significant-high relative bioavailability (172.06 ± 0.14%) over the conventional formulation [24]. Shehata formulated the acemetacin loaded PNs and depicted 85.94% entrapment efficiency as well as sustained drug release. They also exhibited 2.8-fold higher bioavailability than pure acemetacin [25]. Bomma formulated candesartan loaded PNs using maltodextrin. It exhibited high EE (83.24%) and significant-high release (90.1%) compared to pure candesartan [26]. Maltodextrin is used as the inner material for converting the PNs into PNs powder. Maltodextrin is a non-toxic solid material, has better water solubility and provides ease of hydration. Therefore, it is exploited as a carrier to improve entrapment efficiency by enhancing the surface area of hydration [27]. Cholesterol is used in PNs to improve the rigidity of structures, which enhances the drug entrapment efficiency and also improves the stability of the lipid bilayer membrane [28].

The current research work focuses on developing and optimizing esomeprazole PNs powder to enhance the oral bioavailability and ulcer protection activity. The three-factor three levels Box–Behnken statistical design software was utilized to optimize the formulation and evaluated for the vesicle size, surface morphology, EE (%), in vitro drug release, ex vivo permeation, pharmacokinetic and in vivo pharmacodynamic parameters.

## 2. Materials and Methods

### 2.1. Materials

Esomeprazole (EZL) was procured as a gift from Hetero Laboratories Ltd. (Hyderabad, India). Span 60 and cholesterol were purchased from S.D. Fine Chemicals (Mumbai, India), maltodextrin (250 μm average particle size) was purchased from Sigma Alderich (Hyderabad, India), and methanol, and chloroform were purchased from Merck specialties Pvt. Ltd. (Mumbai, India). Other chemicals used in this research work were of analytical grade.

### 2.2. Method

#### 2.2.1. Box-Behnken Statistical Design

Three factors and three levels of Box Behnken design (Design expert version 9.0.1) were employed for optimization of EZL-PNs [13]. The concentration of span 60 (X_1_), cholesterol (X_2_) and EZL (X_3_) were selected as independent variables. The vesicle size (nm, Y_1_), entrapment efficiency (%, Y_2_) and drug release (%, Y_3_) were taken as responses (Table 1). A total of 15 formulations with three centers (same composition) were obtained from the software (Table 2). A 3D response plot and polynomial equation of each response was generated to evaluate the effect of independent variables over the response. The statistical analysis of each model and ANOVA for the best-fitted model was calculated. The optimum formulation was selected as per the desirability function value.

#### 2.2.2. Preparation of Proniosomes

The slurry method was used for the development of PNs using maltodextrin as carrier as shown in Table 2. In brief, the precise amounts of EZL, span 60, and cholesterol in various combinations were taken into a round bottom flask and dissolved in 20 mL of organic solvent (chloroform and methanol 2:1). The required quantity of maltodextrin (200 mg) was added to the above mixture. The organic phase evaporated by placing the round bottom flask on a rotary flash evaporator (Hei-VAP advantage/561-01300, Heidolph, Germany) at 40 °C under reduced pressure. After removing the solvent completely, the PNs powder was subsequently dried overnight in a vacuum oven (Yechem, Sanghai, China). EZL-PNs powder was packed in a closed air tight container and stored at 4 °C for further evaluation [29].

#### 2.2.3. Vesicle Size and Zeta Potential Measurement

A malvern particle size analyzer (Malvern ZetaSizer NanoZS90, Worcestershire, UK) was employed for the determination of vesicle size and zeta potential. The EZL-PNs powder sample was dispersed in deionized water and further diluted 100-fold with deionized water and vortexed. The sample was filled in cuvettes and the vesicle size was analyzed at a 90° scattering angle in triplicate [29]. For zeta potential determination, the diluted EZL-PNs dispersion was filled into a zeta potential cuvette (electrode cuvette) and analyzed in triplicate.

#### 2.2.4. Drug Entrapment Efficiency 

The EE (%) of EZL in EZL-PNs was determined by the centrifugation method. 10 mL of EZL-PNs suspension was filled into a centrifugation tube and settled into a temperature-controlled centrifuge (Beckman, model TJ-6 along with a refrigeration unit, UK). The centrifuge was rotated at 15,000 rpm for 30 min at 4 °C. The supernatant was removed and the pellet was collected. The pellet was dissolved in a mixture of organic solvent (50:50 *v*/*v*), and absorbance was measured using a UV-spectrophotometer at 305 nm [30]. The % EE was calculated by the following formula:

$$\%\;\mathrm{Entrapment}\;\mathrm{efficiency}=\frac{\mathrm{Total}\;\mathrm{EZL}-\mathrm{EZL}\;\mathrm{in}\;\mathrm{residue}}{\mathrm{Total}\;\mathrm{EZL}}\times 100$$

#### 2.2.5. Thermal Transition Analysis

Differential scanning calorimetry (DSC, Mettler Toledo, OH, USA) was used for the determination of thermal spectra of EZL, maltodextrin and the EZL-PNsopt powder. The sample was packed in aluminum crucibles and an empty crucible taken as a blank. The samples were scanned between 25–400 °C at 10 °C/min heating rate with a continuous supply of nitrogen at 20 mL/min [30].

#### 2.2.6. X-ray Diffraction Study (XRD)

XRD spectra of EZL, maltodextrin and EZL-PNsopt were recorded by XRD instruments (Ultima IV diffractometer, Rigaku Inc., Tokyo, Japan). The sample was placed into sample holder in a thin layer. The sample was scanned between a 10–80° angle with 0.5°/min scanning speed. The instrument was operated at 40 kV voltage and a 40 mA current. The diffractograms were captured and compared to each other.

#### 2.2.7. Surface Morphology

A scanning electron microscopy (Carl Zeiss AG-EVO^®^ 50) analysis was done to determine of surface morphology EZL-PNs powder. The optimized EZL-PNs formulation was speckled over an aluminum stub utilizing double-sided adhesive carbon tape, and removed the air under a vacuum. A further sample was coated with gold for 60 s with the Leica Em SCD0050 sputter coater to achieve 14 nm of thickness and scanned under a magnification power ranging between 50×–4k× [19].

#### 2.2.8. In-Vitro Release of EZL

The in vitro release of EZL from EZL-PNs powder was analyzed using USP type-II dissolution test apparatus (Hanson Research, SR8 plus, Hanson, MA, USA). Five hundred mL of release media (0.1 N HCl) was filled in a dissolution basket and the temperature was maintained at 37 ± 0.5 °C. EZL-PNs powder and pure EZL (equivalent to 5 mg of EZL) was dispersed in deionized water and filled into previously soaked cellulose dialysis tubes (MWCO 12000 D, Sigma Aldrich, Bengaluru, India). The edges of the dialysis tube were tightly bound and attached with a paddle of the dissolution apparatus. The paddle was rotated at 50 rpm during entire study. Five mL of aliquot at different time intervals was taken and the same volume of fresh buffers was added simultaneously to maintain the concentration gradient. The absorbance was measured by a UV-Visible spectrophotometer (3200, LABINDIA, Thane, India) at 305 nm. The % cumulative drug release was calculated using Microsoft Excel and plotted the graph between % cumulative drug release and time [26].

#### 2.2.9. Ex Vivo Permeability Study

This study was conducted using the fresh excised rat intestine. The rat was kept in a fasted state for 24 h before sacrifice. The rat was sacrificed and the small intestine (ileum) was collected immediately and cleaned with Krebs solution. The one end of the intestine was tightly tied and the other end was kept open. The EZL-PNsopt and pure EZL dispersions were filled (25 mg of EZL) into the intestine and closed. Then, the intestine was immersed in 200 mL of Krebs solution as permeated media with the continuous supply of air (95% O_2_), and was maintained at a temperature of 37 ± 0.5 °C during the whole study. At definite time intervals, the 2 mL of aliquot was withdrawn and the same volume of fresh buffer was added simultaneously to maintain the concentration gradient. The absorbance was analyzed by UV-spectrophotometry at 305 nm after the appropriate dilution of each sample. The percentage of permeation, flux and enhancement ratio were calculated [25].

#### 2.2.10. In-Vivo Study

An in vivo study (pharmacokinetic and pharmacodynamic) was done on a Wistar albino rat model. The study protocol was approved by the Institutional Animal Ethical Committee of the School of Pharmacy, Anurag University, Hyderabad, India. The ethical approval number of the study is (I/IAEC/AGI/004/2021 WR). The rats (180–200 g, male) were acquired from the central animal house and kept in a department animal house in 12 h dark and light condition and free to eat of food and drinking water.

#### 2.2.11. Pharmacokinetic Study

Wistar albino rats were used for the pharmacokinetic study of EZL-PNsopt and pure EZL dispersion. The rats were divided into two groups, i.e., group-1 for EZL-PNsopt dispersion and group-2 for pure EZL dispersion. EZL-PNsopt and pure EZL dispersion (10 mg/kg body weight) were administered orally using an oral feeding tube. At a definite time (0.5, 1, 2, 3, 4, 6, and 12 h), 1 mL of blood was collected from the retro-orbital vein into the EDTA tube. The blood was centrifuged at 4000 rpm for 15 min and plasma was separated. The EZL was extracted from plasma by the liquid extraction method. Plasma was mixed with formic acid (2% *v*/*v*) and vortexed. Two mL of ethyl acetate was added into the above mixture, vortexed and centrifuged at 4000 rpm for 15 min (4 °C) using a cooling centrifuge (Thermo Fisher Scientific, Mumbai, India). The supernatant was collected and dried in a vacuum oven. The dried extract was dissolved in methanol and filtered through a membrane filter (0.45 µm). The EZL concentration was then determined by the previously validated HPLC method [31]. 20µL of sample was injected into the HPLC column. Ammonium dihydrogen phosphate buffer (0.02 M) and methanol (30:70% *v*/*v*) were used as mobile phase at 1 mL/min flow rate. The plasma concentration vs time graph was prepared and the pharmacokinetic parameters i.e., 1/2 (h), Cmax (ng), Tmax (h), AUC0-t (ng. h/mL) Ke (h^−1^), AUC0-∞ (ng. h/mL), AUMC0-t (µg.h2 /mL), AUMC0-∞ (µg.h2 /mL) were calculated using PK-excel.

#### 2.2.12. In Vivo Pharmacodynamic Study

The in vivo antiulcer activity of EZL-PNsopt was evaluated on Wistar male albino rats and compared with pure EZL. A gastric ulcer was induced by oral administration of a single dose of indomethacin (30 mg/kg body weight) [32]. The rats were kept in a fasting state for 24 h before the experiment. To prevent coprophagy, the rats were kept separately in wide mesh wire bottoms cages throughout the fasting period. The rats were divided into four groups. Group-1 as normal control, Group-2 as disease control, Group-3 as treatment with pure EZL dispersion, and Group-4 received EZL-PNsopt dispersion. The pure EZL and EZL-PNsopt (equivalent to 10 mg/kg body weight) were administered in the treatment group of rats (group-3 and group-4) orally using an oral feeding tube before the administration of indomethacin [33]. Rats were sacrificed by decapitation after 4 h. The stomach was separated and cut from the greater curvature region of the stomach and cleaned with normal saline. The measure of the lesion size was done under three-fold magnification. Ulcer index (UI) and percentage of ulcer protection were calculated by the given equations:

$$\mathrm{Ulcer}\;\mathrm{Index}=\frac{10}{X}$$

$$X=\frac{\mathrm{total}\;\mathrm{mucosal}\;\mathrm{area}}{\mathrm{Total}\;\mathrm{ulcerative}\;\mathrm{area}}$$

$$\mathrm{Percentage}\;\mathrm{of}\;\mathrm{ulcer}\;\mathrm{protection}=\frac{\mathrm{UI}\;\mathrm{of}\;\mathrm{control}-\mathrm{UI}\;\mathrm{of}\;\mathrm{test}}{\mathrm{UI}\;\mathrm{of}\;\mathrm{control}}\;\times \;100$$

#### 2.2.13. Statistical Analysis

The experimental data are represented as mean ±SD. Student’s *t*-test and ANOVA applied using “Graph pad prism (InStat 7; San Diego, CA, USA) for statistical analysis. A *p* < 0.05 was considered significant.

## 3. Result and Discussion

### 3.1. Optimization and Validation

**Figure 1:**
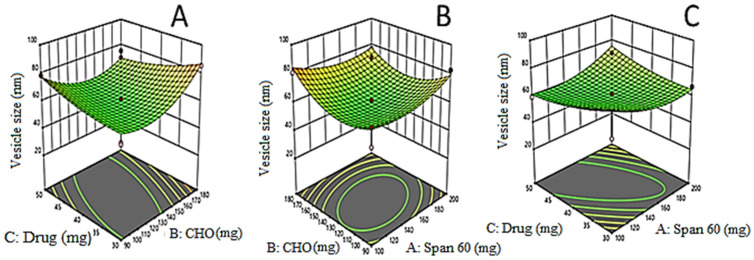
(**A**–**C**): 3D-Response surface plots demonstrating the influence of independent variables i.e., amount of span 60 (X_1_), amount of cholesterol (X_2_) and amount of drug (X_3_) on VZ (nm) of EZL-PNs.

**Figure 2:**
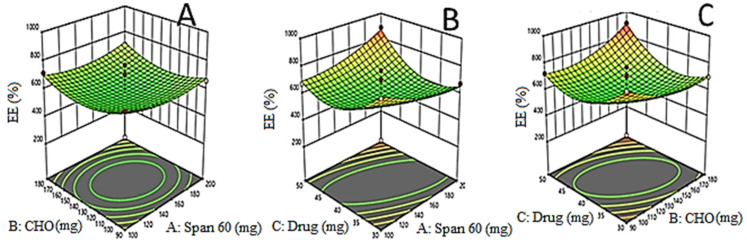
(**A**–**C**): 3D-Response surface plots demonstrating the influence of independent variables i.e., amount of span 60 (X_1_), amount of cholesterol (X_2_) and amount of drug (X_3_) on EE (%).

**Figure 3:**
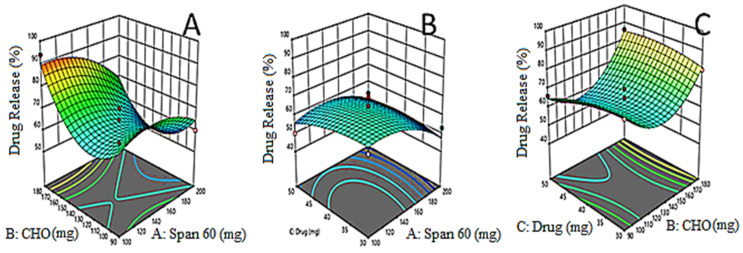
(**A**–**C**): 3D-Response surface plots demonstrating the influence of independent variables i.e., amount of span 60 (X_1_), amount of cholesterol (X_2_) and amount of drug (X_3_) on drug release.

The EZL-PNs powder formulation was optimized by a three-factor, three-level Box Behnken experimental design. EZL-PNs were formulated by a slurry method using span 60 and cholesterol and maltodextrin as carriers. The effect of span 60 (X_1_), cholesterol (X_2_) and EZL (X_3_) was taken as independent variables and their distinct effect was analyzed on vesicle size (Y_1_ nm), EE (%, Y_2_) and DR (% Y_3_). 15 EZL-PNs formulation compositions were obtained with three centre points (same composition). The data of all responses were fitted into different design models i.e., linear, 2 F1, and quadratic. The quadratic model was found to be best for all responses because the regression coefficient R^2^ was found to be maximum. The statistical analysis (R^2^, adjusted R^2^, predicted R^2^, adequate precision, CV, and percentage deviation) of each response were expressed in Table 3. The mathematical relations were recognized and generated coefficients of the second-order polynomial equation for each response. The 3D graph (Figure 1, Figure 2 and Figure 3) of each response was constructed, which explained the effect of multiple independent variables over individual responses. The lack of fit of the best fit quadratic model for each response was found to be non-significant (*p* > 0.05) indicating that the model is well fitted. The optimized formulation of EZL-PNs was selected from a centre point as per minimum vesicle size while maximizing the drug EE and DR.

### 3.2. Effects of Formulation Variables on Vesicle Size (Y_1_)

The vesicle size of the PNs exhibited a definite relationship with the independent factor, i.e., the amount of span 60 (X_1_, % *w*/*v*), cholesterol (X_2_) and drug (X_3_). The individual and collective effect of these independent variables is represented by the polynomial equation:

Vesicle size (Y_1_) = 735.00 + 80.12X_1_ − 39.25X_2_ − 19.63X_3_ − 6.00X_1_X_2_ − 146.75X_1_X_3_ − 23.00X_2_X_3_ − 7.12X_1_^2^ − 80.13X_2_^2^ − 103.63X_3_^2^

The quadratic model was found to be the best fit model for vesicle size with a model F-value is 54.34. The lack of fit was found to be insignificant (*p* > 0.05), which revealed that the quadratic model was well fitted. The 3D surface plot was constructed, which expressed the influence of independent variables on vesicle size (Figure 1A–C). Vesicle size of all EZL-PNs formulations was found to be in the range of 250 ± 12.36 nm (EZL-PNs12) to 905 ± 13.21 nm (EZL-PNs2) as shown in Table 2. Here, the surfactant (span60, X_1_) gives the positive effect on vesicle size, which means increasing the concentration of span 60 and the vesicle size of PNs increased due to increases in the hydrophobicity (HLB 4.7) i.e., saturated long alkyl chain of surfactant [34]. It was also reported that preparing noisome with span 60 as surfactant lead to an increased vesicle (717 nm) [35]. It was further confirmed that low surfactant amount forms thin film and gets hydrated efficiently, forming a smaller vesicle size compared to the high concentration of surfactants. The cholesterol gives the positive effect on vesicle size. The increase in cholesterol and the increase in vesicle size of PNs was observed. Because it increases the width of the bilayer, it hindered the lipid bilayer because the polar head of the cholesterol moves towards the aliphatic chain of the surfactant [34]. Higher cholesterol content is attributed to larger vesicle size (>100 nm). Yoshioka et al. reported that by increasing the cholesterol concentration, an increase in thickness as well as rigidity of the bilayer membrane takes place. It also reduced their fluidity by lessening the vesicle phase transition temperature peak [35].

### 3.3. Effects of Formulation Variables on Drug Entrapment Efficiency (Y_2_)

The effect of independent variables over EE of EZL in EZL-PNs was expressed by mathematically by polynomial equation,

EE (Y_2,_ %) = 75.41 + 1.30X_1_ + 1.98X_2_ + 4.65X3 − 0.5050X_1_X_2_ + 7.37X_1_X_3_ + 14.16X_2_X_3_ + 8.60X1^2^ − 13.47X_2_^2^ − 3.01X_3_^2^

The quadratic model is the best fitted model for EE and model F-value is of 84.21. The lack of fit of quadratic is insignificant (0.05) and revealed that the model is a good fit. The regression analysis is expressed in Table 3. The influence of independent variables on EE is displayed by 3D response surface plots (Figure 2A–C). The % EE of all EZL-PNs was observed to be in the range of 27.92–91.16%. It was found that as the concentration of span 60, cholesterol (CHO) and drug increases, the EE (%) of EZL also increases. CHO increased the EE due to increase the rigidity of lipid bilayer and prevented the leakage of the drug [36]. In addition, Span 60 concentration increases the EE of EZL in proniosomes increases due to highest phase transition temperature (T °C) [37].

### 3.4. Effects of Formulation Variables on Drug Release (Y_3_)

Mathematically the relationship of independent variables with the drug release of EZL from PNs is expressed by polynomial equation:

Drug release (Y_3_) = 58.33 − 3.00X_1_ − 6.75X_2_ − 0.25X_3_ + 0.25X_1_X_2_ + 9.25X_1_X_3_ − 8.75X_2_X_3_ − 1.29X_1_^2^ + 8.71X_2_^2^ + 5.21X_3_^2^

The equation showed the X_1_ (span 60), X_2_ (cholesterol) and (X_3_) drug concentration have a negative effect on drug release. The model F-value is high, which (245.32) indicated that quadratic model fitted well (R^2^ = 0.9943). The *p* = 0.5421 for lack of fit indicated non-significance and it is a good fit for the quadratic model. The adequate precision is greater than four (65.34), indicating that it is a good model. The 3D image (Figure 3A–C) graph displays the influence of the independent variables’ drug release. The drug release from all PN formulations was found to be in the range of 50 ± 2.7% and 94 ± 2.2%. On increasing span 60 and cholesterol concentration, the release of EXL decreases because it reduces the permeability and leakage of formulation. A similar type of finding was reported in flurbiprofen loaded noisome [38].

The present investigation proved that a positive application of a computer optimization technique for the development of a PNs drug delivery system in which amount of maltodextrin, concentration of cholesterol and span 60 significantly effects the vesicle size, EE and DR.

### 3.5. Point Prediction Optimization

**Figure 4:**
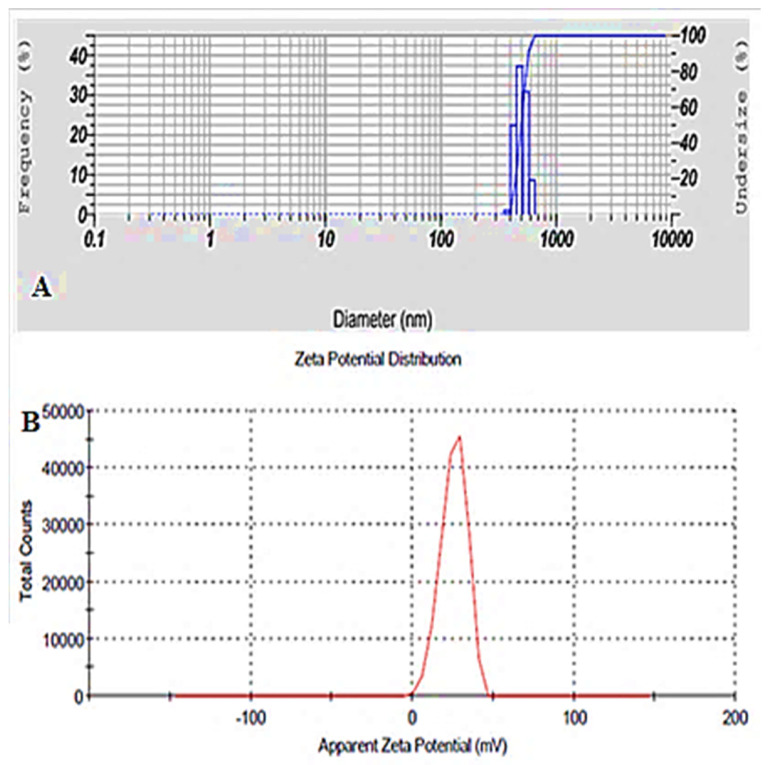
(**A**) Vesicle size and (**B**) Zeta potential graph of EZL-PNsopt.

The optimized formulation (EZL-PNopt) was selected from the point prediction method based on minimum vesicle size, maximum EE and DR [39]. Upon studying various response variables, the optimized formulation (EZL-PNopt) has composition i.e., span 60 (180.05 mg), cholesterol (109.80 mg), amount of drug (40 mg), and maltodextrin (200 mg). The experimental value was found as vesicle size of 616 ± 13.21 nm (Figure 4A), EE of 81.21 ± 2.35% and DR of 95.07 ± 2.10%. The predicted value of vesicle size was found to be 621 nm, EE of 80.24% and DR of 94.23%. The results showed less variation between the predicted and experimental value, indicating the selected variables are well designed.

### 3.6. Vesicle Size, Zeta Potential and Surface Morphology

**Figure 5:**
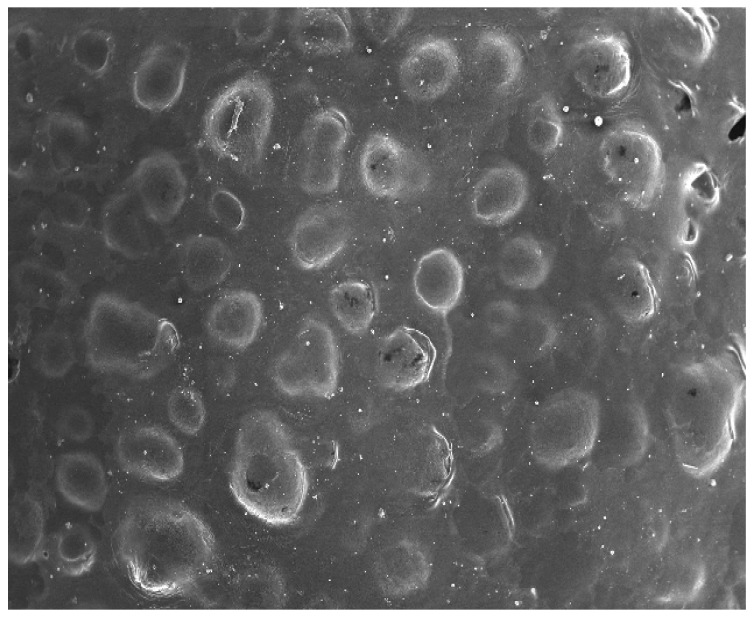
SEM photograph of EZL-PNsopt.

The vesicle size range of EZL-PNs is 250 ± 12.36 nm (EZL-PNs12)–905 ± 13.21 nm (EZL-PNs2) (Table 2)**.** The zeta potential of the EZL-PNsopt formulation was determined and found to be +25.6 mV (Figure 4B). The high value of the zeta potential indicates high stability due to the increased repulsive interaction and stabilization of vesicles with uniform size distribution [19,40]. The surface morphology of EZL-PNsopt powder was analyzed by SEM and the image depicted in Figure 5. The vesicle of PNs exhibited a spherical shape and no formation of drug crystal or aggregation was observed. It could be easily re-dispersed in water [11].

### 3.7. Entrapment Efficiency (EE %)

The EE of EZL within the different prepared EZL-PNs formulations was found in the range of 27.92 ± 2.06% and 91.16 ± 2.35% (Table 2). The EE of EZL-PNsopt was found to be 81.21 ± 2.35%. The high EE is due used span 60 among different grades of spans which is attributed to the long alkyl chains present in span 60 [41].

### 3.8. Thermal Transition Analysis

**Figure 6:**
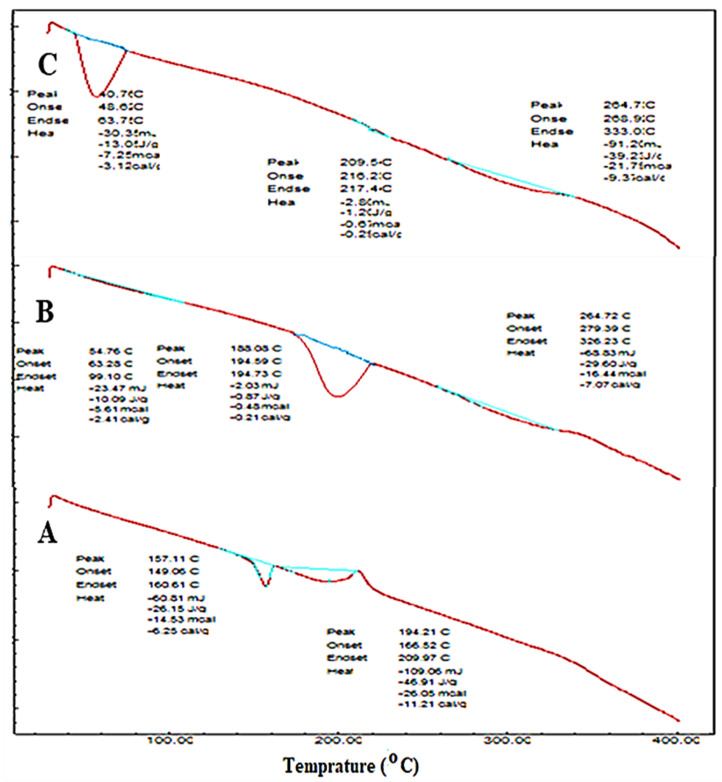
Thermal transition analysis of (**A**) pure EZL, (**B**) maltodextrin and (**C**) EZL-PNsopt.

Figure 6 explained the DSC thermogram for pure EZL, maltodextrin and the EZL-PNsopt formulation. The spectra of EZL showed the characteristic endothermic peak at 157.11 °C (Figure 6A), and it is corresponding melting point of EZL. The maltodextrin exhibited a broad endothermic peak at 194.73 °C (Figure 6B). The peak of EZL was missing in EZL-PNopt powder (Figure 6C), demonstrating that the drug was encapsulated or solubilized into the lipid matrix [19].

### 3.9. X-ray Diffraction Study

**Figure 7:**
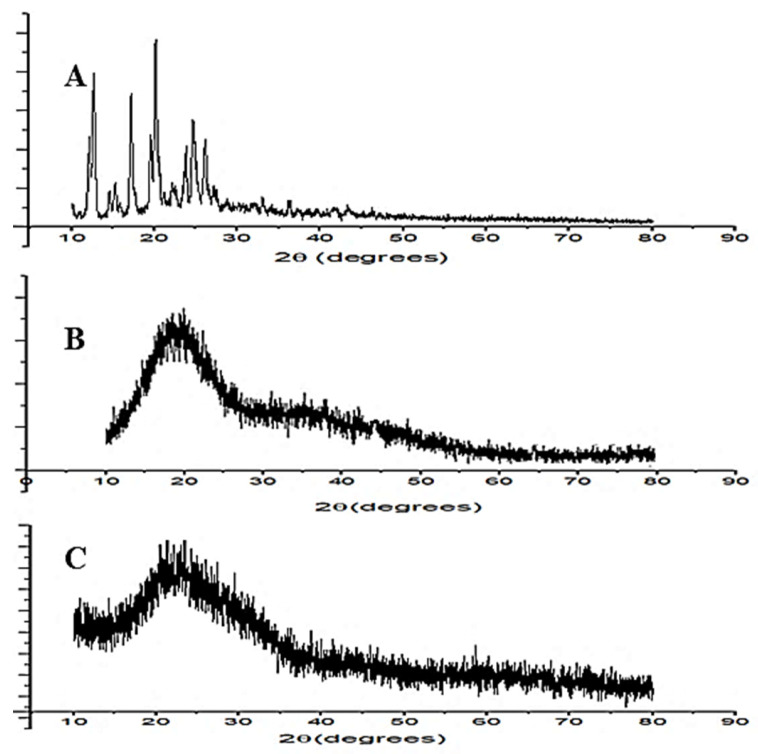
X-ray diffraction spectra of (**A**) pure EZL, (**B**) maltodextrin and (**C**) EZL-PNsopt.

Figure 7 depicts the XRD-spectra of pure EZL, maltodextrin and EZL-PNsopt formulation. The XRD-spectra of EZL exhibited the characteristic intense peaks at 12.2°, 17.4°, 20.2°, 25.2°, and 26.8° at 2theta level (Figure 7A) assuring that crystalline nature of EZL. The spectra of maltodextrin did not show any crystalline peaks (Figure 7B). However, the EZL-PNsopt formulation does not exhibit any characteristic peaks of EZL in their spectra (Figure 7C). It revealed that EZL was encapsulated or dissolved in the PNs matrix. A similar type of result was observed in the PNs of valsartan [42].

### 3.10. In Vitro Release of EZL

**Figure 8:**
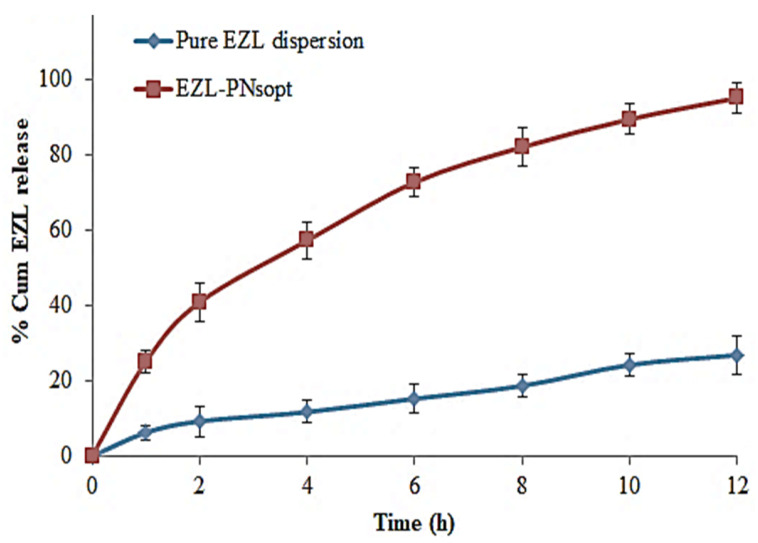
In vitro drug release profile of EZL-PNopt and pure EZL.

The percentage cumulative EZL release from EZL-PNsopt and pure EZL dispersion was analyzed by using a dialysis bag, and the results showed in Figure 8. A significant high (*p* < 0.05) release was found from EZL-PNsopt as compared to pure EZL dispersion. The EZL-PNs exhibited a dual release pattern, an initial fast release (57.21 ± 2.9 % in 4 h), followed by prolonged-release (95.07 ± 4.4%, 12 h). The initial fast release could be attributed to free non-entrapped EZL and/or adsorbed EZL on the surface of PNs. The slow-release is due to the release from the PNs matrix. The high release of EZL from PNs is due to the presence of surfactant which decreases the interfacial tension and leads to better wettability [43]. However, the pure EZL dispersion exhibited only 26.81 ± 3.1% release in 12 h due to poor aqueous solubility.

**Figure 9:**
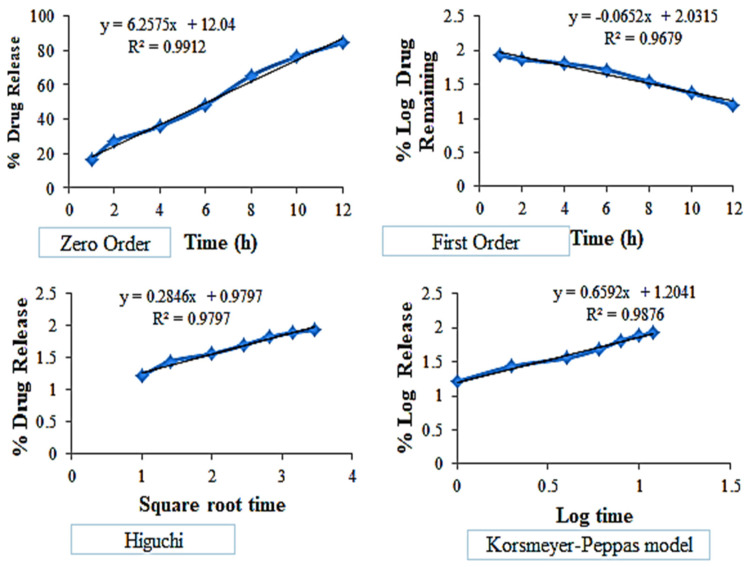
In vitro release kinetic model for EZL-PNsopt.

The release profile of the EZL-PNsopt was fitted into various kinetic models i.e., the zero-order, first-order, Higuchi and Korsmeyer Peppas model, and data was depicted in Figure 9. The zero-order model was found to be the best fit because it has a maximum regression co-efficient value, (R^2^ = 0.9911) indicating that the concentration was independent of the drug release.

### 3.11. Ex Vivo Intestine Permeation Study

**Figure 10:**
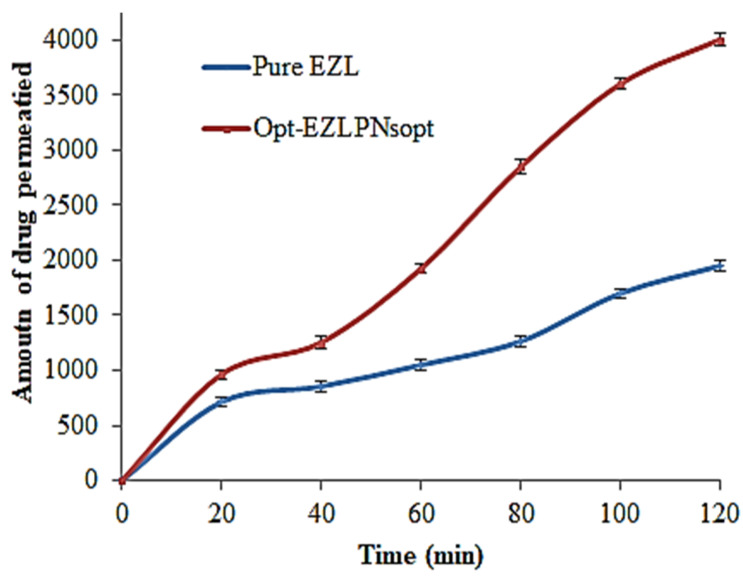
Ex-vivo gut EZL permeation profile from pure EZL and EZL-PNsopt.

The amount of drug permeated (ADP) across the rat intestine was determined and the results are expressed in Figure 10. The cumulative amount of EZL permeated across the intestine was found to be 1949.50 ± 1.1 µg from pure EZL, and 4000.22 ± 1.8 µg from EZL-PNsopt, respectively. Flux of EZL-PNopt was found to significantly (*p* < 0.05) higher (33.684 µg/cm^2^.min) than pure EZL (14.69 µg/cm^2^.min). The enhancement ratio from EZL-PNsopt was found to be 2.24-fold higher than pure EZL dispersion. The flux of the drug is attributed to the direct transfer of the drug from the vesicle system to the absorption site [42]. This release behavior of PN justified the significance of vesicular systems. It seems that PNs can serve as a penetration enhancer by modulating the lipids and increasing the fluidity [29].

### 3.12. Pharmacokinetic Studies

**Figure 11:**
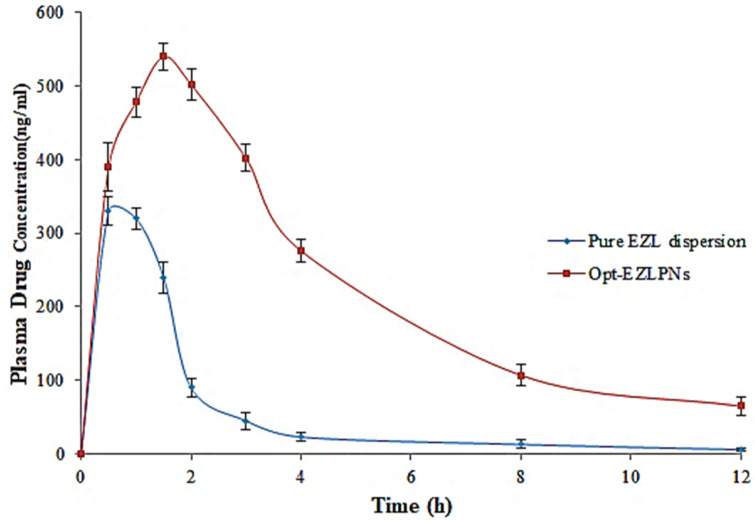
EZL plasma concentration vesicle size time profile of pure EZL and EZL-PNsopt.

The pharmacokinetic study of EZL from EZL-PNsopt and pure EZL were done on rats, and data was calculated using PK excel sheet software. The PK parameters such as t_1/2_ (h), C_max_ (ng), T_max_ (h), AUC_0-t_ (ng. h/mL), Ke (h^−1^), AUC_0-∞_ (ng. h/mL), AUMC_0-t_, (µg.h^2^/mL), AUMC_0-∞_ (µg.h^2^/mL) values were determined. The mean plasma concentration–time profiles of EZL for the EZL-PNs-opt and pure EZL is shown in Figure 11, and data of pharmacokinetic parameters express in Table 4. The t_1/2_, C_max_, T_max_ AUC_0-t,_ Ke, AUC_0-∞_, AUMC_0-t_, AUMC_0-∞_ of EZL-PNs-opt are 3.27± 0.16 h, 540 ± 18 ng, 1.5 ± 0.04 h, 2730.5 ± 8.45 ng. h/mL, 0.211 h^−1^, 3038.03 ± 19 ng. h/mL, 10444.5 ± 24 ng.h^2^/mL and 15589.91 ± 19 ng.h^2^/mL, respectively. However, t_1/2,_ C_max_, T_max_ AUC_0-t,_ Ke, AUC_0-∞_, AUMC_0-t_, AUMC_0-∞_ of pure EZL are 1.35 ± 0.12 h, 330 ± 20 ng, 0.5 ± 0.03 h, 679 ± 12.05 ng. h/mL, 0.351 h^−1^, 696.08 ± 21(ng. h/mL), 1482.5 ± 23 ng.h^2^/mL and 1736.11 ± 25 ng.h^2^/mL respectively. The EZL-PNs-opt showed an increase in the half-life and a lesser elimination constant due to the slow release of EZL as compared to pure EZl. The relative bioavailability of EZL-PNopt was found to be 4.02-fold compared with pure EZL. It concluded that PNs formulation of EZL-PNopt has demonstrated increased the solubility of the drug, eventually enhancing its absorption [44].

### 3.13. In Vivo Pharmacodynamic Study

**Figure 12:**
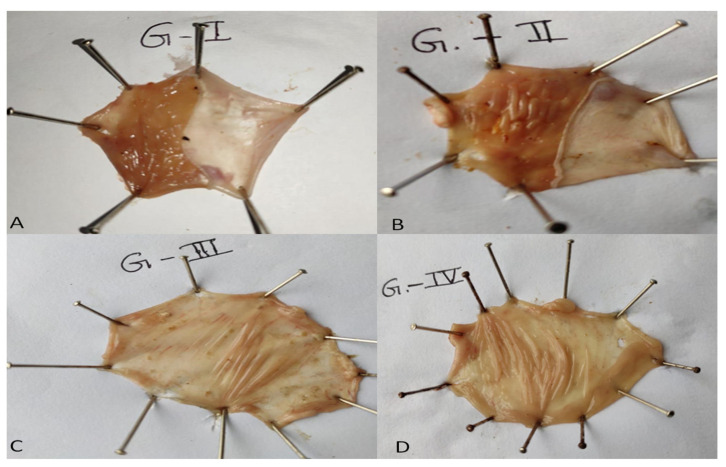
Photographs of (**A**) non-ulcer induce stomach (Group-I), (**B**) indomethacin induced (Group-II), (**C**) Pure EZL (Group III) (**D**) EZL-PNopt (Group IV).

Figure 12 exhibited the antiulcer activity of pure EZL and EZL-PNopt. The ulcer index was found to be significantly increased after oral administration of indomethacin (Group-2), which may be linked to either the formation of free radicals or the suppression of prostaglandin synthesis [36]. Low levels of prostaglandin are linked to impaired gastro-protection and enhanced gastric secretion, which are significant events causing mucosal ulceration. The ulcer index value was found to be 1.02 ± 0.1 for the control group, (Group-I), 7.85 ± 0.13 for Group-II, 4.41 ± 0.3 for group-III (pure EZL dispersion), and 3.01 ± 0.1 for Group-IV (EZL-PNopt), respectively (Table 5). The EZL-PNsopt formulation exhibited a significantly (*p* < 0.05) lower ulcer index than pure EZL. The percentages of ulcer protection of pure EZL and EZL-PNsopt after administration was found to be 43.82% and 61.65%, respectively. This report is in agreement with previously reported research by Bendas and his associates [43]. Indomethacin-induced gastric lesions are stasis in gastric mucosa, which promotes the growth of hemorrhagic and necrotic characteristics of the tissue injury [45]. The in vivo anti-ulcer assessment showed that the EZL-PNopt formulation was able to diminish ulcer formation produced by the oral administration of indomethacin.

## 4. Conclusions

The optimization and development of EZL-PNs is a novel area of research that exploits the appealing properties of nano vesicular systems to enhance the delivery of active molecules. EZL-PNs was successfully developed by the slurry method using span 60, cholesterol, and maltodextrin, and optimized by box Bhekhen statistical design software. The developed EZL-PNs exhibited nano-size, positive zeta potential and spherical morphology. The solid-state characterization (DSC and XRD) revealed that EZL was encapsulated into the lipid bilayer of the PNs matrix. EZL-PNsopt exhibited high entrapment efficiency (81.21 ± 2.35%) and sustained release (95.07 ± 2.10% in 12 h) as well as significant high intestinal permeation (4000.22 ± 1.8 µg) compared to pure EZL (1949.50 ± 1.1 µg). A pharmacokinetic study demonstrated EZL-PNsopt’s significantly high relative bioavailability (4.02-fold high) compared with pure EZL. A pharmacodynamic study of EZL-PNsopt showed significant high ulcer protection (61.65%) compared to pure EZL (43.82%). The study concluded that developed PNs could be suitable carriers for delivery of EZL for the improvement of bioavailability and ulcer protection activity.

## Figures and Tables

**Table 1:** Process variables and their levels for experimental design.

Factors	Levels Used, Actual Coded		
**Independent Variables**	**Low (−1)**	**Mid (0)**	**High (+1)**
X_1_ = Span 60 (mg)	100	150	200
X_2_ = Cholesterol (mg)	90	135	180
X_3_ = Drug amount (mg)	30	40	50
**Dependent Variables**	**Goals**		
Y_1_ = Vesicle size (nm)	Minimize		
Y_2_ = Entrapment efficiency (%)	Maximize		
Y_3_ = Drug release (%)	Maximize		

**Table 2:** Composition of esomeprazole loaded maltodextrin-based proniosomes powder.

Formulation Code	Span 60 (X_1_)	Cholesterol (X_2_)	Drug (X_3_)	Vesicle Size (Y_1_, nm)	Entrapment Efficiency (Y_2,_ %)	Drug Release (Y_3,_ %)
EZL-PNs1	1	1	0	723 ± 12.32	78.57 ± 4.56	66 ± 2.1
EZL-PNs2	−1	−1	0	905 ± 13.21	75.38 ± 3.13	86 ± 1.1
EZL-PNs3	0	0	0	660 ± 12.16	82.73 ± 3.44	91 ± 2.4
EZL-PNs4	0	−1	1	698 ± 11.18	85.59 ± 4.68	80 ± 2.6
EZL-PNs5	0	1	1	704 ± 11.11	76.93 ± 1.98	65 ± 1.1
EZL-PNs6	1	0	1	771 ± 13.71	61.62 ± 1.98	70 ± 1.2
EZL-PNs7	1	−1	0	848 ± 12.40	59.37 ± 1.09	75 ± 2.4
EZL-PNs8	1	0	−1	889 ± 12.10	70.82 ± 3.38	53 ± 2.3
EZL-PNs9	0	0	0	667 ± 10.81	81.65 ± 2.35	93 ± 2.1
EZLPNs10	−1	0	−1	720 ± 10.91	70.32 ± 2.86	73 ± 2.2
EZL-PNs11	−1	0	1	674 ± 12.92	66.82 ± 0.42	53 ± 2.4
EZLPNs12	−1	1	0	250 ± 12.36	27.92 ± 2.06	56 ± 2.3
EZL-PNs13	0	1	−1	715 ± 13.21	80.85 ± 2.05	80 ± 2.2
EZL-PNs14	0	0	0	663 ± 11.09	81.23 ± 0.63	92 ± 2.7
EZL-PNs15	0	−1	−1	825 ± 12.31	80.85 ± 2.05	62 ± 2.8

**Table 3:** Model statistical summary of quadratic mode for vesicle size, EE and DR.

Source	Vesicle Size	Entrapment Efficiency	Drug Release
Model	Quadratic	Quadratic	Quadratic
Adjusted R^2^	0.9920	0.9521	0.9819
R^2^	0.9934	0.9656	0.9865
Predicted R^2^	0.9813	0.9543	0.9802
% CV	3.03	3.87	5.65
Adequate precision	65.05	28.43	43.87
Standard Deviation	6.87	3.14	3.23

**Table 4:** Pharmacokinetic parameters of pure EZL dispersion and EZL-PNopt. The parameters evaluated on six animals and shown as mean ± SD.

Pharmacokinetic Parameter	Pure EZL Dispersion	Opt-EZLPNs
t_1/2_ (h)	1.35 ± 0.12	3.27 ± 0.16
C_max_ (ng)	330 ± 20	540 ± 18
T_max_ (h)	0.5 ± 0.03	1.5 ± 0.04
AUC_0-t_ (ng. h/mL)	679 ± 12.05	2730.5 ± 8.45
Ke (h^−1^)	0.351	0.211
AUC0_-∞_ (ng. h/mL)	696.08 ± 21	3038.03 ± 19
AUMC_0-t_ (µg. h^2^/mL)	1482.5 ± 23	10,444.5 ± 24
AUMC0_-∞_ (µg. h^2^/mL)	1736.11 ± 25	15,589.91 ± 19

**Table 5:** Anti-ulcer activity of the EZL-PNopt formulation-treated group compared to other groups.

Group	Ulcer Index %	Ulcer Protection %
Control (Group-I)	1.02 ± 0.1	**-**
Indomethacin Induced (Group-II)	7.85 ± 0.13	**-**
EZL-Standard (Group-III)	4.41 ± 0.3	43.82
EZL-PNopt (Group-IV)	3.01 ± 0.12	61.65

## Data Availability

Not applicable.

## Sample Availability

Samples of the compounds are not available from the authors.
